# Development of the Cardiac Conduction System and the Possible Relation to Predilection Sites of Arrhythmogenesis

**DOI:** 10.1100/tsw.2008.40

**Published:** 2008-03-03

**Authors:** M. R. M. Jongbloed, E. A. F. Mahtab, N. A. Blom, M. J. Schalij, A. C. Gittenberger-de Groot

**Affiliations:** ^1^ Department of Anatomy and Embryology of the Leiden University Medical Center, Leiden, The Netherlands; ^2^ Department of Cardiology of the Leiden University Medical Center, Leiden, The Netherlands; ^3^ Department of Paediatric Cardiology of the Leiden University Medical Center, Leiden, The Netherlands

**Keywords:** cardiac conduction system development, arrhythmias, electrophysiology, pulmonary veins, cardiac development

## Abstract

The cardiac conduction system (CCS) encompasses a complex system responsible for the coordinated contraction of the heart. In the developing heart, as well as in the adult heart, tissues of the (putative) CCS are characterized by different properties than the surrounding working myocardium, which can be observed on a histological level, as well as by the expression patterns of several immunohistological and molecular markers. In recent years, many markers have been studied that have helped to elucidate the processes involved in CCS development. It has become clear that multiple genes, cells and their interactions are involved in this complex process. In this article, an overview of the current knowledge of CCS development is supplied. Furthermore, several controversies regarding conduction system development are discussed, as well as the possible significance of embryologic development of the CCS for the development of arrhythmias later in life.

## INTRODUCTION

Cardiac arrhythmias are frequently encountered in clinical practice. Clinical mapping studies demonstrate that arrhythmias are often found at specific anatomical sites. The development of the heart and the cardiac conduction system (CCS) cannot be seen as loose entities, but are closely related (Fig. 1). Therefore, in this article, cardiac development will be described shortly, after which the development of the CCS will be addressed. Subsequently, anatomical predilection sites for the occurrence of clinical arrhythmias will be described in relation to CCS development.

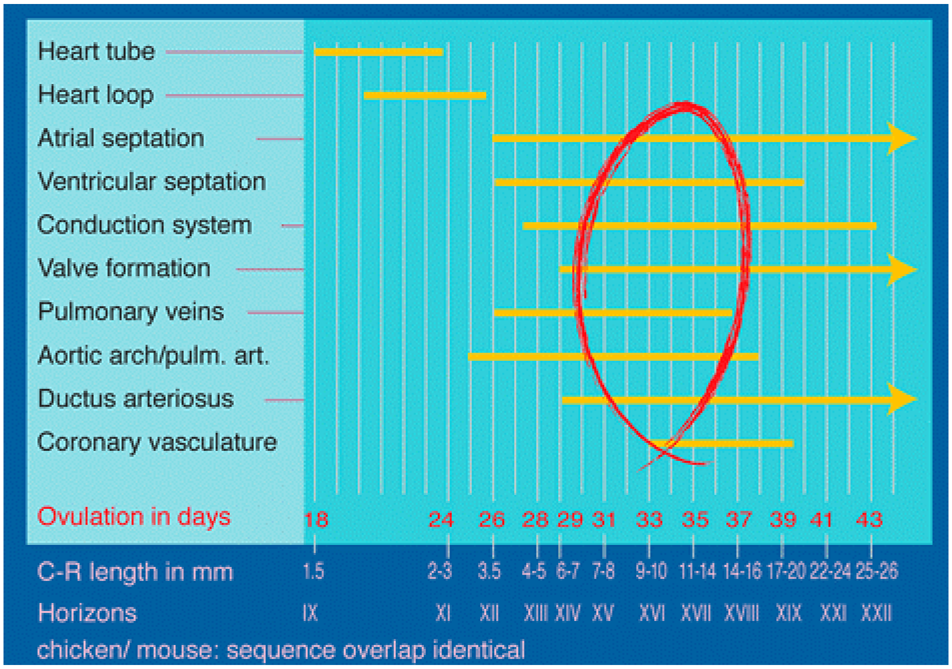

**FIGURE 1.** Schematic overview of the time span of development of the different cardiac components. Development of the CCS is narrowly related to the development of the cardiac chambers and vascular system.

## SHORT OUTLINE OF CARDIAC DEVELOPMENT

In vertebrates, the heart is the first organ that is formed and becomes functional during early embryogenesis. Future cardiac cells arise in the epiblast lateral to the primitive streak, invaginate through the streak, and migrate during gastrulation in a rostrolateral direction to bilateral areas of the lateral plate mesoderm[1,2,3]. The lateral mesoderm separates in somatic and splanchic epithelial layers; the (asymmetric)[4,5,6] bilateral splanchic mesoderm contains the cardiac precursors cells (the primary heart field) that migrate toward the embryonic midline and fuse to form the primary myocardial heart tube, lined on the inside with endocardium that is separated from the myocardial outer layer by cardiac jelly[7,8] (Fig. 2a–c). During further development of the heart tube, cells from the splanchic mesoderm (the second heart field) will continue to contribute to the heart. This contribution takes place at both the arterial and venous poles of the heart (Fig. 3). The contribution from cells to the dorsal (venous) pole of the heart is described by several research groups[9,10,11,12,13,14] and we have particularly referred to this contribution as derived from the posterior heart field[10]. Progenitor cells in the pharyngeal mesoderm, referred to as the anterior[15] and secondary[16] heart field, will contribute to the arterial pole and right ventricle of the heart. The heart tube is initially attached to the embryonic (noncardiac) mesoderm via the dorsal mesocardium, which is disrupted during looping, only leaving contact at the arterial and venous pole. After looping, the heart tube consists of several segments: the left and right horn of the sinus venosus, the primitive atrium, the ventricular inlet segment, and the ventricular outlet segment. These segments are divided by so-called transitional zones that connect at the inner curvature of the heart (Fig. 2d)[17].

Chamber differentiation occurs during further rightward looping of the heart tube, which results in positioning of the ventricles and the outflow tract of the heart in an anterior/ventral position, and of the atria in a dorsal/posterior position. Several genes/transcription factors that control chamber differentiation have been identified[18,19].

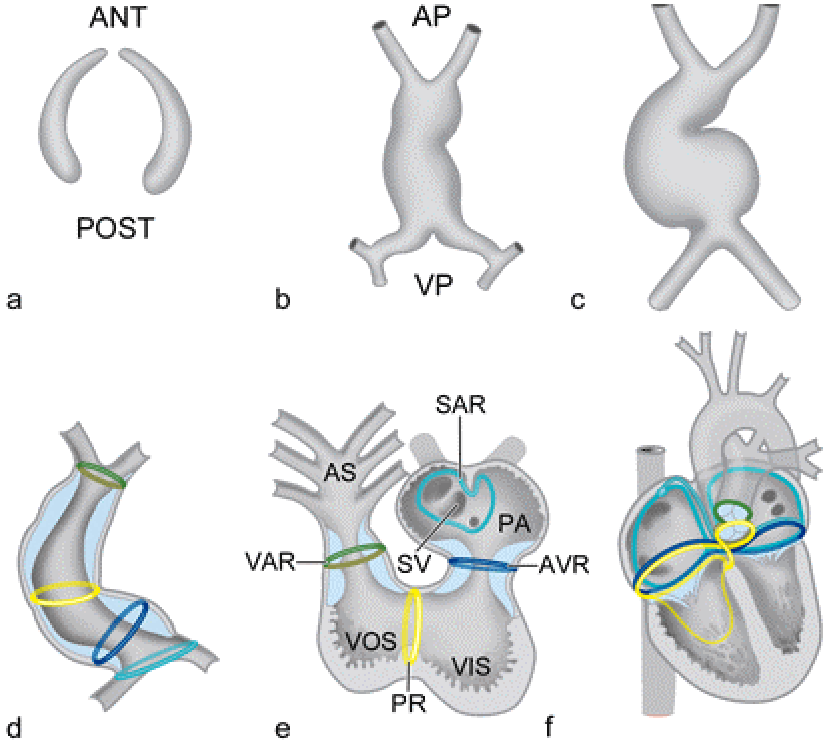

**FIGURE 2.** Schematic representation of the bilateral formation of the cardiogenic plates, which are derived from the splanchnic mesoderm (a). The bilateral plates fuse and form an initially straight heart tube (b) that starts looping to the right (c,d). After looping, so-called transitional zones or rings can be recognized in the heart that are positioned in between the putative cardiac chambers, i.e., the sinoatrial transition (SAR), the atrioventricular transition (AVR), the primary ring (PR), and the ventriculoarterial transition (VAR) (e). Position of these rings during further cardiac development (f). Ant: anterior, AP: arterial pole, AS: aortic sac, PA: primitive atrium, post: posterior, SV: sinus venosus, VIS: ventricular inlet segment, VOS: ventricular outlet segment, VP: venous pole. Panel a–c adapted from Gittenberger-de Groot et al.[5]

The sinus venosus becomes incorporated in the atrium and receives the venous inflow of the cardiac veins. The cardiac jelly becomes more concentrated at the atrioventricular (AV) junction and the outflow tract, sites where endocardial cushions and, subsequently, cardiac valves and membranous septa will form. Extracardiac neural crest cells migrate to the heart and play an important role in outflow tract septation[20,21]. Myocardialization of the walls of the caval and pulmonary veins presumably results from myocardium formation at the venous pole of the heart[10,11,22], also referred to as the posterior heart field[10], although migration of cardiomyocytes from the atrial wall has also been postulated[23]. Migration of cardiomyocytes from the atrium may also contribute to myocardialization of the coronary veins[24]. Cardiac septation and the formation of valves at the right and left AV junctions, and in the right and left ventricular outflow tracts, eventually results in the presence of a functional four-chambered heart that directs the separate systemic and pulmonary circulation of blood.

## DEVELOPMENT OF THE CARDIAC CONDUCTION SYSTEM

The origin of the cells of the CCS has been the topic of interest of many studies in the last decade. The cells of the CCS were originally believed to be derived from the cardiac neural crest, a migratory cell population that arises at the embryonic neural plate at the level of the otic placode[25,26]. However, retroviral reporter gene transfection lineage studies have demonstrated that *cardiomyocytes* are the progenitors of the cardiac

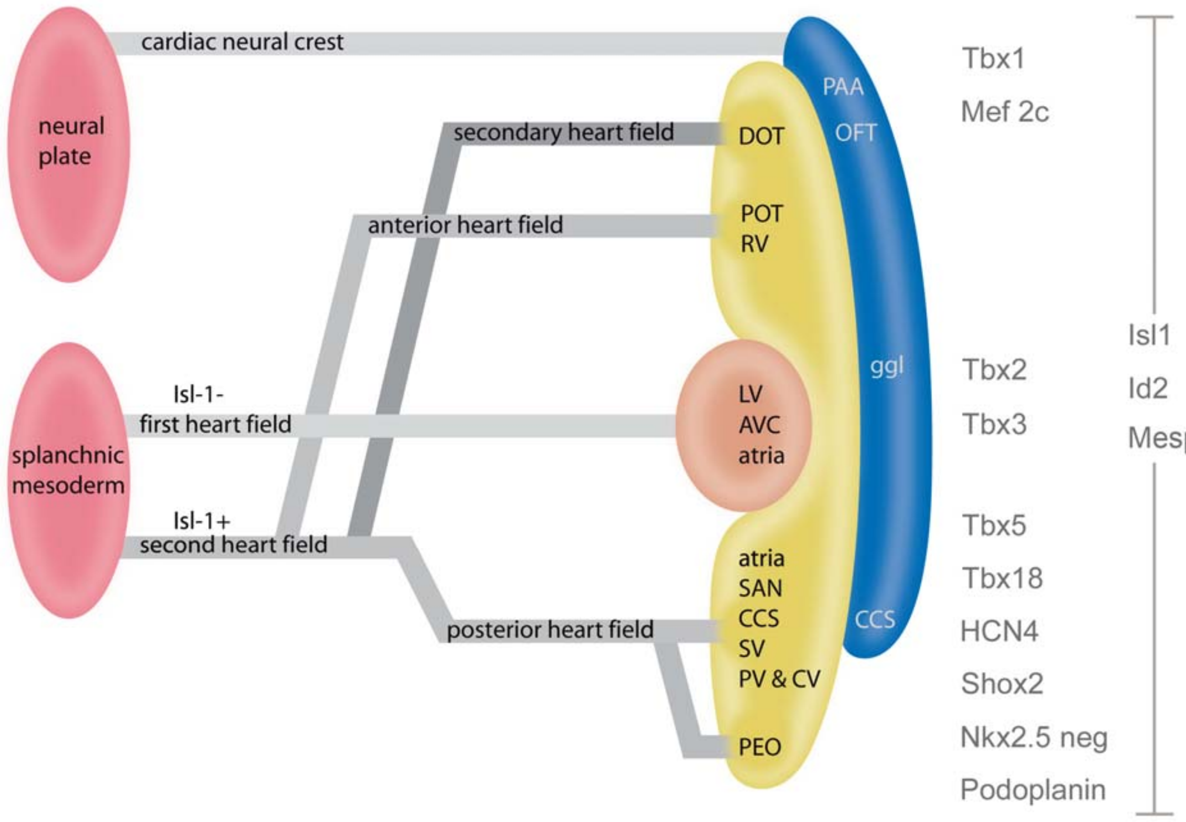

**FIGURE 3.** Schematic representation of the primary heart tube (brown) and the secondary added myocardium derived from the second heart field (yellow), including differential genes and proteins expressed in the second heart field. The second heart field can be divided into an *anterior heart field* and a *secondary heart field* at the anterior pole of the heart, and a *posterior heart field* at the venous pole of the heart. At the venous pole of the heart, the proepicardial organ (PEO) is also derived from the posterior heart field, and is the source of the epicardium and epicardium-derived cells. Neural crest cells (depicted in dark blue) migrate to the heart and enter the heart at both the arterial and venous pole. AVC: atrioventricular canal, CV: cardinal veins, CCS: central conduction system, DOT: distal outflow tract, LV: left ventricle, OFT: outflow tract, PAA: pharyngeal arch arteries, POT: proximal outflow tract, PV: pulmonary veins, RV: right ventricle, SAN: sinoatrial node, SV: sinus venosus.

conduction cells in the embryonic heart[27]. Whether the cardiomyocytes that form CCS tissue are derived from the division of differentiated (prespecified) conduction cells (*"specification-model"*) or are recruited from a pool of multipotent (undifferentiated) cardiomyogenic cells (*"recruitment-model"*) is, however, still unclear[28]. The origin of the CCS is also viewed by some researchers as myocardium originating from the primary heart tube, in which the expression of certain genes prevents differentiation of this myocardium to a chamber-working myocardium phenotype. In this theory, this first heart field–derived myocardium will contribute to the CCS, while the chamber myocardium balloons out from the primary heart tube (the so-called ballooning model)[29,30] (Fig. 3).

Studies performed in chick embryos have demonstrated the development of both working myocardial cells and central and peripheral conduction cells from the same clone, and therefore strongly indicate that cells of the CCS originate from a common myogenic precursor in the embryonic tubular heart, i.e., a *bipotential myocardial cell population* that is selectively recruited to the developing cardiac pacemaking and conduction system[27,31]. However, the mechanisms that determine the fate of the cardiomyocytes to become either a working myocardial cell or a cardiac conduction cell are still unclear. The growth and differentiation factor Neuregulin can induce cardiomyocytes to a conduction system phenotype, as was demonstrated by the induction of ectopic CCS-*lacZ* expression after exposure to Neuregulin[32]. Furthermore, changes in electrical activation patterns supporting a critical role of Neuregulin in recruitment of cardiomyocytes to the cardiac pacemaking and conduction system have been observed. Recently, an interaction between Endothelin and Neuregulin has been suggested to promote the differentiation of the murine CCS[33]. Gassanov et al. describe differentiation of atrial-derived cardiomyocytes to a pacemaker-like phenotype induced by Endothelin-1 (but not Neuregulin), using murine embryonic stem cells expressing enhanced green fluorescent protein (EFGP) under the transcriptional control of the ANP (atrial natriuretic peptide) promotor[34].

## EXTRACARDIAC CONTRIBUTIONS TO THE CARDIAC CONDUCTION SYSTEM

### Neural Crest Cells

Neural crest cells migrate to the heart and enter the heart at two sites. The population of cardiac neural crest cells that enters the heart via the *arterial pole* contributes to the formation of the aorticopulmonary septum and outflow tract endocardial cushions[35,36]. Another population of neural crest cells enters the heart at the *venous pole* and can be observed in the vicinity of putative elements of the CCS before they undergo a fate of apoptosis, which has led to the hypothesis that these cells may indirectly be involved in CCS differentiation[37,38,39] (Fig. 3). Recent work from Gurjarpadhye et al. demonstrates that neural crest ablation in chick results in lack of differentiation of the compact lamellar organization by the His bundle (that separates it from the working myocardium)[40]. Nakamura et al., who also demonstrated the presence of neural crest cells near elements of the CCS in mice, describe that some of these neural crest–derived cells possess glial markers that are known to be expressed in cells contributing to the electrical insulation of nerves[41].

### Epicardium-Derived Cells

The second extracardiac contribution to the heart comes from cells that are derived from the epicardium and, therefore, are named epicardium-derived cells (EPDCs). Epicardium formation is initiated by the formation of the proepicardial organ, a villous structure that protrudes in the pericardial cavity close to the venous pole of the heart[36] (Fig. 3). As new molecular data indicate that this structure is also derived from the posterior heart field, the true extracardiac origin of the EPDC can be debated[10,36].

EPDCs contribute to several cardiac structures, including the coronary arteries, the AV valves, the fibrous heart skeleton, and the myocardial architecture[36]. In the CCS, EPDCs are important for the induction of Purkinje fiber formation[42]. The peripheral Purkinje fibers develop from differentiating ventricular cardiomyocytes[27], in close association with both the coronary arteries and EPDCs[27,43,44]. Inhibition of proepicardial outgrowth causes Purkinje fiber hypoplasia and abnormal differentiation of Purkinje fibers in quail embryos[42]. EPDCs may either be involved in Purkinje fiber development by cooperation with inducing factors secreted by endothelial and endocardial cells, or by production of endothelial factors themselves[36,45].

In a recent study, both epicardium and EPDCs were found to express periostin, which is found in colocalization with EPDCs in the AV valves and fibrous heart skeleton, also contributing to the annulus fibrosis that electrically isolates the atria from the ventricles[46].

### *Histology*

Although in recent years several immunohistochemical and molecular markers of the developing CCS have been identified, the original descriptions of the developing CCS are based on strictly histological criteria as observed with light microscopy. Thirty years ago, Viragh and Challice described in great detail the developing CCS in mouse embryos[47,48,49,50]. From these studies, it has become clear that areas of putative CCS can be distinguished from the working myocardium based on histological criteria. In these studies, cells of the developing CCS were characterized by a larger cell size, less developed and reduced number of myofibrils, and a higher glycogen content than working cardiomyocytes.

The earliest sign of a morphologically specialized AV conduction pathway can be observed at embryological day (E) 9–10 in the mouse, and is located at the inner dorsal wall of the AV canal[47]. During development, this primordial AV node becomes structurally more compact. At stage E11, the primordia of both the sinoatrial node (in the medioanterior wall of the right superior caval vein) and the AV node can clearly be distinguished[49]. Both these structures, as well as the AV bundle, develop simultaneously in the mouse heart, between E11–E12 (5–5.5 weeks in the human). At E13.5, all components of the CCS can be distinguished, with the exception of the Purkinje fibers.

The AV node is connected with the His bundle that is located on the ridge of the interventricular septum[50]. The left and right bundle branches extend down the subendocardial layers on both sides of the interventricular septum.

Insulation of the atrial myocardium from the ventricular myocardium occurs by development of the annulus fibrosis, which starts out by fusion of sulcus tissue with cushion tissue at the ventricular site of the AV junctional myocardium and moves the original AV myocardium to an atrial position[51]. At E13–14, connective tissue begins to invade the AV sulcus and histological separation of the atria from the ventricles is initiated. Also, progressive insulation between the cells of the CCS and the ventricular working myocardium occurs. These processes, however, remain incomplete until birth and continue in the neonatal period[50,52].

Some authors have described an anterior AV node related to congenital heart disease[53,54]. A dual origin of the AV node is supported by observations with the marker HNK1 in humans[55]. In the histological studies by Viragh and Challice, also a left-sided sinoatrial node was observed in the medial wall of the left superior caval vein, which eventually became integrated into the dorsal wall of the left atrium[49]. A dual sinoatrial node is a condition that has been associated in humans with right isomerism[56,57]. Most recent observations that are based on the expression pattern of the coelomic and myocardial marker podoplanin in the heart contribute a role for the posterior heart field in the myocardial Anlage of a transient left-sided sinoatrial node and the definitive right sinoatrial node[10], suggesting a bilateral development of the sinoatrial node. Interestingly, the earliest pacemaker in the tubular heart was demonstrated at the site of the *left* atrium primordium[58]. Whether these cells also contribute to the AV conduction system, as postulated[10], remains to be proven.

Another recent study indicates that the transcription factor *Pitx2c*, involved in left/right signaling in the heart, suppresses sinus node formation on the left side[59], as fetuses that lack the expression of *Pitx2c* exhibit right isomerism and form sinus nodes at both the right and left sinoatrial junction[59,60,61].

## The Four-Ring Theory of CCS Development

Using the same histological criteria as Viragh and Challice to distinguish working myocardium from myocardium with more specialized features, the observation was made that after looping of the heart has started, four rings of tissue could be distinguished from the surrounding working myocardium, as described in 1976 by Wenink[62]. These four rings are positioned at the above-described transitional zones of the heart[17]: the *sinoatrial ring,* between the sinus venosus segment and the primitive atrium; the *AV ring,* between the primitive atrium and primitive left ventricle; the *primary ring* or *fold,* that separates the primitive left ventricle from the primitive right ventricle; and the *ventriculoarterial ring,* at the junction of the primitive right ventricle with the truncus or putative outflow tract of the heart (Fig. 2d). At these transitional zones, different staining properties of the myocardium, as well as size and chromatin distribution of the cells, indicated the presence of primitive specialized tissue. The so-called "ring theory" hypothesizes that these four rings of "specialized" tissue are the precursors of the CCS. During further looping of the primitive heart tube, these four rings come together in the inner curvature of the heart (Fig.

2e,f) and during further differentiation of the heart, part of the tissue loses its specialized character. What remains of the rings becomes the definitive elements of the mature CCS. According to this theory, the sinoatrial ring contributes to the formation of the sinoatrial node, both the sinoatrial ring and AV ring contribute to the AV node, and the primary ring gives rise to the His bundle and bundle branches. Since its introduction, this theory has been the subject of discussion and controversy, which was renewed in recent years after the introduction of several immunohistochemical and molecular markers for CCS development.

## *Immunohistochemical Markers of CCS Development*

In the past decades, several immunohistochemical markers have been used to study the developing CCS. Although many of these markers have increased our understanding of CCS development, a limitation is that none of them is specific for CCS only. In the 1990s, the expression pattern of a neurofilament-like protein in the rabbit heart was used as a marker for the developing CCS[26]. The presence of neurofilament-like protein was demonstrated in a ring at the sinoatrial and AV junctions, and in ventricular components of the developing CCS, which were distributed in the ventricular subendocardium and connected to the AV ring[26].

Expression of the monoclonal antibody HNK1, originally used as a marker of neural crest cells during embryologic development, is observed in the sinus venosus myocardium and in the developing CCS of several species, including rats, chicks, and humans[55,63,64]. HNK1 is predominantly expressed in the developing sinoatrial and AV CCS, and the expression pattern seems to correspond with the rings described by Wenink. In human embryos, HNK1 stains the sinoatrial node, the internodal myocardium in the right atrium, the right AV ring with the posterior and anterior AV nodes, a retroaortic ring, the His bundle, and the bundle branches. Furthermore, the myocardium surrounding the primitive pulmonary vein demonstrates transient staining[55] (Fig. 4).

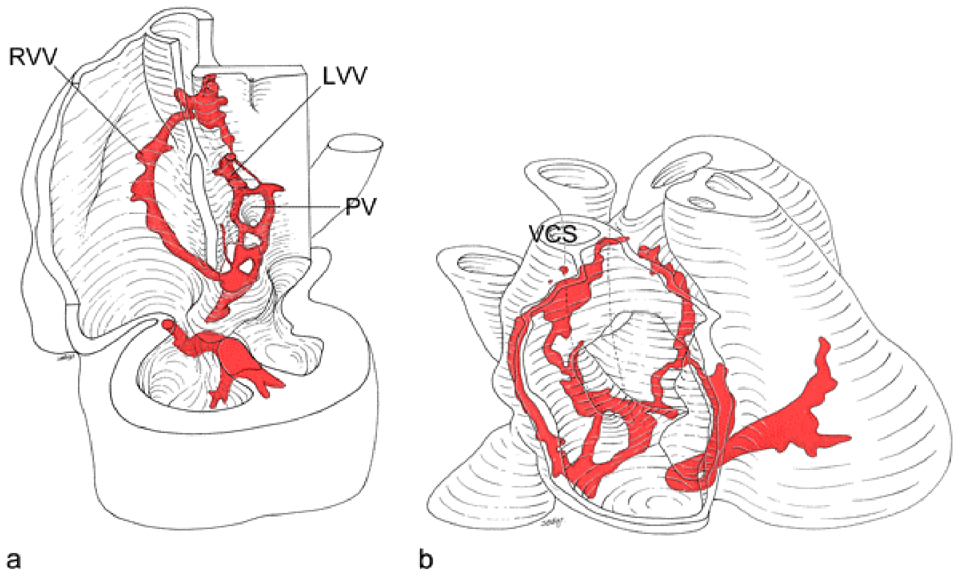

**FIGURE 4.** HNK1 expression in the human embryo. Explanation see text. LVV, left venous valve; PV, primitive pulmonary vein; RVV, right venous valve; VCS, superior caval vein. Adapted from Blom et al.[55]

The neural tissue antigen Gln2, highly homogenous to HNK1, was described to be expressed in a single ring of tissue at the site of the primary ring in the early embryonic heart, which changes shape during development as a result of tissue remodeling underlying cardiac septation. This ring was hypothesized to give rise eventually to the AV CCS[65].

The cell surface carbohydrate PSA-NCAM has been detected in ventricular trabeculae and the interventricular septum in the chick, in a pattern resembling the bundle branches and Purkinje fibers[66].

Recently, we described the expression of podoplanin as a coelomic and myocardial marker[10]**.** Podoplanin is a 43-kd, mucin-type transmembrane glycoprotein that is found outside the heart in, e.g., osteoblasts; the nervous system; epithelia of lung, eye, esophagus, and intestine; mesothelium of the visceral peritoneum; the podocytes of the kidney; and lymphatic endothelium[67,68,69,70]. Expression of podoplanin in the heart indicates this protein as a marker for developing sinus venosus myocardium from the posterior heart field. Podoplanin is expressed in the coelomic lining (in close contact with the sinoatrial nodal myocardium) and in the underlying mesenchyme adjacent to the cardinal veins. Podoplanin-positive mesenchyme differentiates into myocardium that stains negative for Nkx2.5. During cardiac development, podoplanin is expressed in myocardium along the right and left cardinal vein and in both a right-and left-sided sinoatrial node (which persists in later stages only in the right-sided sinoatrial node and part of the venous valves, an expression pattern opposed to Nkx2.5), the base of the atrial septum, the posterior AV canal, the AV nodal region, and the His Purkinje system. Also, during later developmental stages, podoplanin is expressed in the pulmonary veins[10]. In podoplanin$^{-/-}$ mice, myocardialization around the pulmonary veins is diminished, and there is underdevelopment of the atrial septum (E.A.F. Mahtab, A.C. Gittenberger-de Groot et al., in preparation) (Fig. 5).

## *Molecular Markers for CCS Development*

In recent years, extensive study focusing on genetic determinants of CCS formation has evolved. Studies of transcription factors involved in cardiogenesis have made clear that regulation of myocardial differentiation into either a conductional or working myocardial phenotype is not dependent on a single gene, but is a multifactorial process during which several factors from different gene families contribute to the formation of the different subcompartments of this complex system. Molecular markers that have been used to delineate (elements of) the developing CCS include *minK-lacZ*, CCS-*lacZ*, *cGATA-6-lacZ*, *cardiac troponin I-lacZ* , *GATA-1*, the homeodomain transcription factor *Nkx2.5*, the recently described *Hop* and *Shox2*, *Id2*, *HCN4*, and the T-box transcription factors *Tbx2*, *Tbx3*, and *Tbx5*. Furthermore, the expression pattern of several connexins in cardiac tissues has contributed to our understanding of the development and function of the CCS[71]. Most of these transcription factors do not function in an autonomic matter, but interact with other factors, resulting in synergistic or repressing effects. The currently known molecular markers of CCS development are briefly described in the subheadings below.

### The T-Box Family of Transcription Factors

In the developing heart, the T-box transcription factors *Tbx2* and *Tbx3* are expressed in the cardiac inflow tract, the AV canal, the outflow tract, and inner curvature of the heart. These factors presumably are transcriptional repressors of chamber formation, as both genes repress the genes *Nppa* (*ANF*) and *Cx40*, present in (for example) atrial working myocardium[29,72]. In general, expression of *Tbx2* and *Tbx3* is mainly observed in putative, slow-conducting areas, but also in the His bundle and the proximal part of the bundle branches. The expression of *Tbx2* decreases from early fetal stages, whereas the expression of *Tbx3* increases. In the developing heart, expression of *Tbx3* is observed in the sinoatrial node and AV node, but also in internodal myocardium, and in the His bundle and proximal bundle branches[72]. Next to expression in part of the putative CCS, *Tbx3* expression is also observed in the AV cushions. Homozygous *Tbx3* mutant mice display a syndrome known in humans as *ulnarmammary syndrome* and display early embryonic mortality, presumably due to severe compromise of the yolk sac[73]. Recently, the function of *Tbx3* in controlling the sinoatrial node gene program has been described[74]. *Tbx3* is expressed in the developing

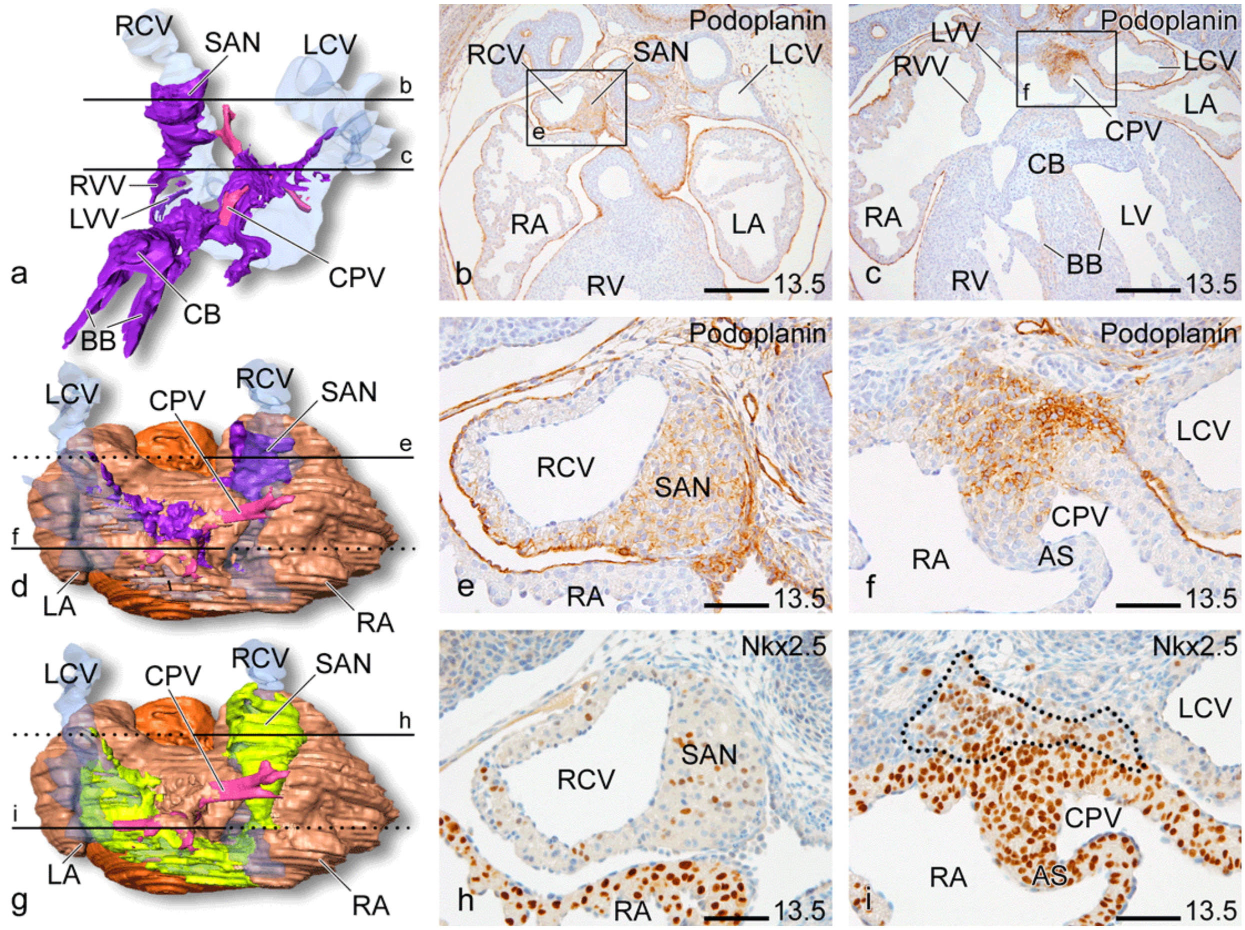

**FIGURE 5.** Three-dimensional (3-D) reconstructions (a, d, g, for color codes see below) and transverse sections (b, c, e, f, h, i) of an E13.5 wild-type (WT) mouse heart demonstrating podoplanin and Nkx2.5 expression. a: Ventral view of a reconstruction giving an overview of podoplanin expression in various parts of the CCS myocardium. *Line b* indicates podoplanin expression in the sinoatrial node region (SAN) and *line c* indicates podoplanin staining around the common pulmonary vein (CPV) corresponding to sections b and c, respectively (for details see e and f). Podoplanin expression is indicated in the SAN, right (RVV) and left (LVV) venous valves, the atrioventricular nodal region (AVN), common bundle (CB) and bundle branches (BB). d and g: dorsal view of reconstructions showing, respectively, podoplanin-positive (purple) and Nkx2.5-negative (lime green) areas at the sinus venosus region of the embryonic heart. *Line e* demonstrates a dorsal view of podoplanin expression at the SAN and *line f* demonstrates a dorsal view of podoplanin expression around the CPV, indicated in sections e and f, respectively (boxed area in b and c). *Lines h and i* indicate similar regions as lines e and f showing Nkx2.5-negative areas of the SAN (section h) and around the CPV (marked area in section i). The podoplanin-positive area of the sinus venosus region corresponds with the more extensive Nkx2.5-negative (mosaic) area (compare d with g). AS: atrial septum, LA: left atrium, LCV: left cardinal vein, LV: left ventricle, RA: right atrium, RCV: right cardinal vein, and RV: right ventricle. Color codes — light brown: myocardium of the atria, dark brown: myocardium of the ventricles, transparent blue: cardinal veins, pink: pulmonary veins, purple: podoplanin-positive myocardium, and lime green: Nkx2.5-negative (mosaic) myocardium. Scale bars — b and c: 200 µm; e, f, h, and i: 100 µm.

and mature sinoatrial node and is required to suppress the expression of genes regulating atrial differentiation. Furthermore, *Tbx3* can induce ectopic pacemaker sites in the atria[74]. The T-box transcription factor *Tbx5* is also expressed in the developing central CCS, including the AV node, AV bundle, and bundle branches, and is needed for correct morphogenesis and maturation of the CCS[75]. Mice lacking *Tbx5* display a cardiac phenotype that resembles the *Holt-Oram syndrome*, including atrial septal defects and conduction system abnormalities[18]. *ANF* and *Cx40*, both expressed in cells of the (fast conducting) CCS, are gene targets of *Tbx5*, and *Cx40* is abrupted in *Tbx5*-mutated mice (*Tbx5*$^{del/+}$)[75]. *Tbx18* is expressed in the sinus horns and is most likely essential for proper formation of the sinus venosus, as in mice deficient for *Tbx18*, formation of the sinus venosus is disturbed[11].

## Homeodomain Transcription Factors

The homeodomain transcription factor *Nkx2.5* is one of the earliest markers of the cardiac lineage and is already expressed in the cardiogenic mesoderm[76]. During cardiac development, expression of *Nkx2.5* correlates with the recruitment of cells to the developing AV conduction system[77]. During development of the CCS, *Nkx2.5* expression is elevated in the differentiating AV conduction system, compared to expression in the adjacent working myocardium. In *Nkx2.5* haploinsufficient mice, there is hypoplasia of the AV node and His bundle, and the number of peripheral Purkinje fibers is significantly reduced[78]. Cardiac phenotypes of mutations in *Nkx2.5* in mouse models resemble those in humans and include conduction defects[79]. Nkx2.5 is not expressed in posterior heart field–derived myocardium, including the sinoatrial node and the sinus venosus[10,11].

Furthermore, *Nkx2.5* interacts with the *Cx40* promoter region and mice lacking *Nkx2.5* demonstrate a significant decrease in *Cx40* expression[80]. *Nkx2.5* can form a complex with the transcription factor *Tbx2* that is able to *suppress ANF* promoter activity in the AV canal, which may be a mechanism that helps to regulate the sites of chamber formation in the developing heart[81]. *Nkx2.5* can also bind to *Tbx5* and both are essential components in the *activation* of the *ANF* gene.

The homeodomain transcription factor *Msx2*, a downstream target of *Pax-3/*splotch (which is a key player within early cardiac neural crest development), is expressed in the developing central CCS, but not the peripheral Purkinje fibers, in the chick. However, no abnormalities in the cardiac conduction have been observed in *Msx2*-mutant mice[82,83].

The homeobox gene *Hop* is strongly expressed in the AV node, His bundle, and bundle branches of the adult CCS and *Hop*-null mice demonstrate conduction defects below the AV node, related to decreased expression of Cx40[84].

The homeodomain transcription factor *Shox2* is expressed in the embryo in the craniofacial region, limbs, brain, and heart[85], In the heart, it can be detected as early as E8.5 in the posterior region of the primitive heart tube. During further development, *Shox2* is expressed in the sinus venosus myocardium, which includes the sinoatrial nodal region and the venous valves, and expression is also observed in the primitive left and right bundle branches[86]. *Shox2* knock-out mice die between 11.5 and 13.5 days postcoitum, and show severe hypoplasia of the sinus venosus myocardium of the posterior heart field, including a decreased size of the sinoatrial node region and hypoplastic venous valves. Interestingly, in knock-out mice, aberrant expression of *Cx40*, *Cx43*, and Nkx2.5 is observed within the sinoatrial node, indicating abnormal differentiation of the sinoatrial node as well as disturbed pacemaker function of the node in zebrafish embryos[86]. Given these results, an important function for *Shox2* in recruiting sinus venosus myocardium, including the sinoatrial nodal region, was hypothesized.

The bicoid-related homeodomain transcription factor *Pitx2c* is involved in directing left/right identity in the heart at the venous pole[60] and is probably involved in suppression of left-sided sinus node formation, as *Pitx2c*-deficient fetuses form sinoatrial nodes in both the right and left atrium[59].

## Id Family of Transcriptional Repressors (Helix-Loop-Helix Containing Transcriptional Repressors)

Recently, conduction system–specific expression of *Id2* was described[87]. The gene *Id2*, identified by serial gene expression analysis (SAGE) as having ventricular conduction system expression, is a downstream target of *Tbx5* and *Nkx2.5*. Specification of the ventricular CCS fails in mice haploinsufficient for both *Tbx5* and *Nkx 2.5*. $Id2^{-/-}$ mice demonstrate ECG features of abnormal *inter*ventricular conduction, such as left bundle branch block in newborn and adult knock-out mice. Furthermore, intracardiac recordings are consistent with abnormal *intra*ventricular conduction within the bundle branches. $Id2^{-/-}$ mice display abnormal morphology of the AV bundle and left bundle branch, similar to abnormalities observed in adult mice with *Tbx5* haploinsufficiency[87]. *In situ* hybridization demonstrated that *Id2,* expressed in the CCS in wild-type hearts, is not expressed in compound$Tbx5^{+/-}$

/*Nkx2.5*$^{+/-}$ hearts, indicating that ventricular CCS-specific expression of *Id2* is dependent on *Nkx2.5* and *Tbx5*[87].

## Basic Helix-Loop-Helix (bHLH) Transcription Factors

Nonexpression of the bHLH transcription factor *Mesp1* has recently been reported in the ventricular conduction system[88].

## The GATA Family of Transcription Factors/Zinc Finger Subfamilies

The GATA-family is a relatively small family of transcription factors, and for three of the six known vertebrate GATA transcription factors, a role in cardiogenesis has been identified: *GATA4*, *GATA5,* and *GATA6*[89]. Expression of *GATA4* is present in both the adult and embryonic heart, and disruption results in cardiac dysmorphogenesis with early embryonic mortality[90]. The large degree of interaction of the different transcription factors is again shown in a recent study that demonstrated that, next to *Tbx3* and *Nkx2.5*, the *Cx40* promoter is also modulated by the cardiac transcription factor *GATA4*[80]. *GATA4* is expressed in Purkinje fibers of the adult chick heart[91]. *GATA5* mRNA is observed in the precardiac mesoderm of the primitive streak embryo. In the embryonic heart, there is expression of the *GATA5* gene in the atrial and ventricular chambers that, during further development, becomes restricted to the atrial endocardium[92]. Furthermore, c*GATA5* is expressed in the endocardial cushions and in the CCS, in the sinoatrial node, AV node, bundle of His, and left and right bundle branches[93]. Interestingly, the *GATA5* gene is also expressed in a dynamic fashion over time in the septum transversum and epicardial organ in the mouse and avian heart, giving rise to the (*GATA5*-expressing) epicardium[93]. The *cGATA6* gene enhancer specifically marks components of the developing AV CCS and AV node[94,95], but not the more distal components of the CCS. Expression of *cGATA6* remains visible in the mature CCS.

## *MinK/lacZ* Knock-In/Knock-Out

The *minK* gene (also known as *IsK* and *KCNE1*) encodes a 129-amino-acid protein that modifies electrical currents in the heart resulting from expression of the genes *HERG* and *KvLQT1*[96]. Mutations in both *HERG* and *KvLQT1* that encode the structural subunits for the channels involved in the cardiac-delayed rectifier currents $I_{Kr}$ and $I_{Ks}$, respectively, are the most common causes of congenital long-QT syndrome (LQTS)[96]. Disruption of the *minK* gene and integration of the *lacZ* gene results in β-galactosidase expression under the control of endogenous minK regulatory elements, which has been used to study the expression pattern of *minK* in mice.

Disruption of the *minK* gene causes inner ear defects and QT interval prolongation in bradycardic conditions, the combination of which is known as the *Jervell-and Lange-Nielsen syndrome*[97]. *MinK*$^{-/-}$ myocytes lack the delayed rectifier current $I_{Ks}$ and demonstrate significantly reduced $I_{Kr}$, which indicates a role of *minK* in modulating both rectifier currents[96]. *MinK-lacZ* is expressed in the developing CCS in murine embryos starting on E8.25[98]. Expression was observed in discrete rings at the sinoatrial, AV, interventricular, and ventriculoarterial junctions, and became more restricted during further development to, for example, the venous valves, and components of the definitive cardiac conduction tissues. Expression was not observed at the site of the pulmonary veins[98].

## CCS-*lacZ* Insertional Mutation

In 2000, fortuitous insertion of a *lac*Z gene in the murine genome unexpectedly resulted in expression of *lacZ* in the (developing) conduction system of the heart. Although the gene was originally referred to as

"*Engrailed2-lacZ*", the transgene is most likely under the transcriptional control of an unidentified integration site and not by the *Engrailed2* regulatory elements included in the transgene proper. The gene was therefore renamed to CCS-*lacZ* by Fishman et al.[32]. Optical mapping studies performed in murine embryos demonstrated a clear correlation of electrical activation with CCS-*lacZ*-expressing areas[32,99]. Study of the genetic background of *CCS-lacZ* expression in this model has shown rearrangement of chromosome 7 between regions D1 and E1, with altered transcription of multiple genes in the D1 region. The same study indicated that regulatory elements from the gene *Slco3A1* influences CCS-restricted reporter gene expression[100]. Members of the Slco family encode for organic anion-transporting polypeptides that mediate transport of both natural substances (such as prostaglandins, bile salts, thyroid, and steroid hormones) as well as exogenous drugs (including digoxin, angiotensin converting enzyme inhibitors, HMG-coenzyme A reductase inhibitors, methotrexate, and rifampin) across the cell membrane[101,102]. With the extent of the recombination observed in the CCS-*lacZ* model, it is likely that regulatory elements from more than one gene may be involved[100] (Fig. 6).

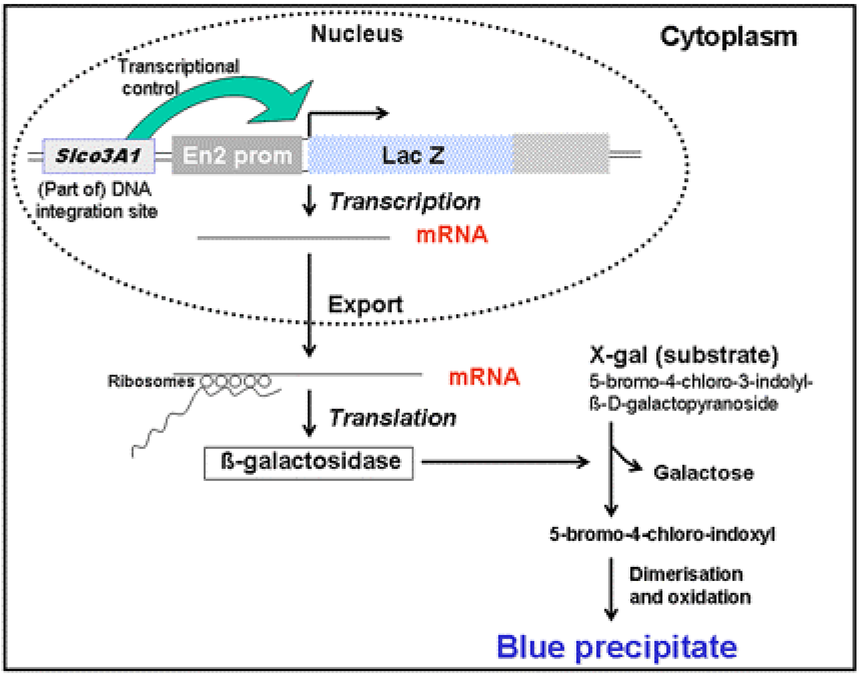

**FIGURE 6.** Simplified working scheme of the transgenic CCS-*lacZ* mouse model. The bacterial *lacZ* reporter gene was placed under the control of engrailed2 promoter elements. Random integration of the construct in the mouse genome resulted in β-galactosidase expression in the cardiac pacemaking and conduction system throughout the heart. β-Galactosidase catalyzes the conversion from X-gal to 5-bromo-4-chloro-indoxyl, which is visible after nonenzymatic dimerization and oxidation as a blue precipitate in the cells in which the reporter gene is expressed. As expression in the CCS was not observed in a number of additional lines of mice harboring the same transgenic construct, it is likely that the *lacZ* expression is under the transcriptional control of an unknown locus at the site of integration, rather than of the *En-2* regulatory elements within the construct. Further study indicated that regulatory elements from the gene *Slco3A1* influence CCS-restricted reporter gene expression.

CCS-*lacZ* is expressed in all components of the developing CCS, including the right and left venous valves and septum spurium of the sinus venosus (Fig. 7) and putative sinoatrial node, the left and right AV ring, His bundle, bundle branches, and Purkinje fibers. CCS-*lacZ* is also expressed in the moderator band of the right ventricle, Bachmann's bundle, the retroaortic root bundle, and in the myocardial sleeve that develops around the pulmonary vein, areas related to arrhythmogenesis in adults. Findings in this model

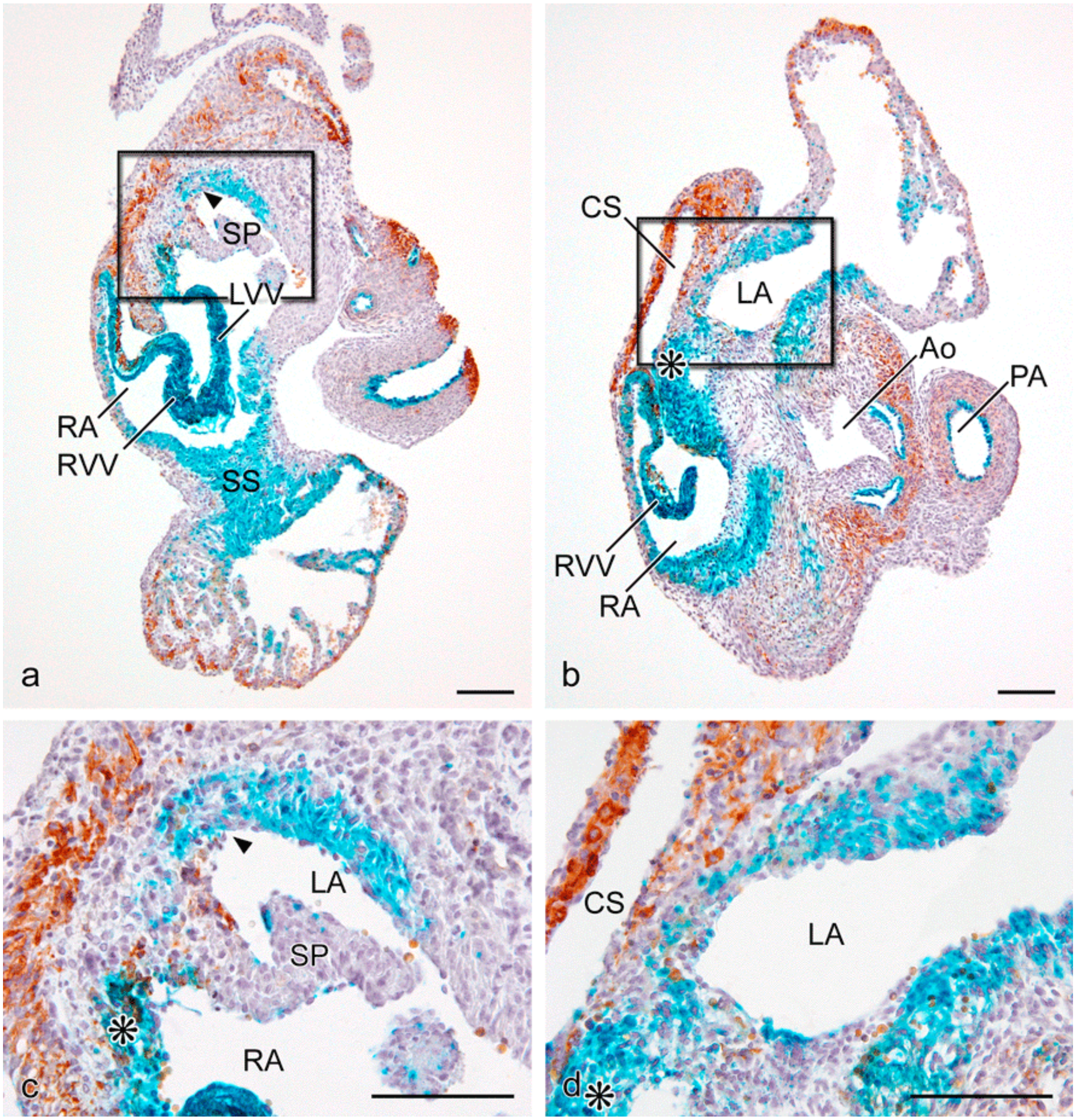

**FIGURE 7.** Transversal sections at the atrial level of a CCS-*lacZ* mouse at age E14.5. a,b: Sections at the level of entrance of the pulmonary vein (arrow head) in the left atrium (a) and through the coronary sinus (b). CCS-*lacZ* expression is observed in the right venous valve (RVV), left venous valve (LVV), and septum spurium (SS) of the sinus venosus. c,d: Details of the boxed areas in a and b that demonstrate continuity of the CCS-*lacZ*–positive myocardium of the left atrial dorsal wall with the base of left venous valve of the sinus venosus (asterisk) in the right atrium. Ao: aorta, CS: coronary sinus, LA: left atrium, PA: pulmonary artery, RA: right atrium, SP: septum primum. Adapted from Jongbloed et al.[104]. Scale bars:100 µm.

thus supported the hypothesis that the occurrence of cardiac arrhythmias in the heart is not random, but may be related to persisting, cq-reactivated areas of developing CCS[103,104,105].

CCS-*lacZ* expression can also be observed in intraluminal endothelial cells, which are hypothesized to be linked to the secretion of endothelial-derived factors involved in induction of cardiomyocytes to acquire a conduction system phenotype. Indeed, the endothelial paracrine factor Neuregulin-1 has been demonstrated to induce ectopic expression of CCS-*lacZ* and therefore may play a critical role in recruitment of cells to the CCS[32]. Timing of exposure to the endothelial factors may be crucial, as the inductive effect of Neuregulin in the CCS-*lacZ* mouse was restricted to a window of sensitivity between E8.5 and E 10.5[32].

In the adult mouse heart, using serial sections of CCS-*lacZ* hearts, Cx40 immunostaining (marking ventricular CCS cells) could be colocalized with CCS-*lacZ* transgene expression in the AV node, His bundle, bundle branches, and subendocardial Purkinje fibers along the interventricular septum[106]. In contrast to the developing heart and neonatal heart, in the adult mouse heart, CCS-*lacZ* expression can no longer be demonstrated within the sinoatrial node[107].

### The Hyperpolarization-Activated Cyclic Nucleotide-Gated Cation (HCN) Channel Family

Four genes that encode HCN channels have been identified: *HCN1, HCN2, HCN3, and HCN4*. HCN channels carry an inward current, the depolarizing Na/K current $I_f$, that underlies cardiac pacemaker activity. In the adult heart, both *HCN2* and *HCN4* are expressed. During development, *HCN4* is expressed as early as E7.5 in the cardiac crescent[108,109]. Interestingly, in the early heart tube (E8), expression is observed bilaterally in the sinus venosus, corresponding to previous optical mapping studies by Kamino et al. and studies in the chick by van Mierop et al.[58,110]. Later in development, expression of *HCN* becomes asymmetrical and restricted to the right atrium, at the site of the developing sinoatrial node[108]. In the postnatal and adult heart, *HCN4* is highly expressed in the sinoatrial node[108,109]. *HCN4* knock-out mice die between E 9.5 and E 11.5. As these knock-out mice do not display mature pacemaker potentials, it is likely that *HCN4* channels are required for proper pacemaker function of the sinoatrial node[109]. The expression pattern of *HCN4* overlaps with the expression of markers of the posterior heart field, such as podoplanin and *Shox2*[10,86]. The expression of *HCN4* reflects the sinus venosus myocardium of the posterior heart field and becomes restricted to the sinoatrial node[108].

*HCN2* is expressed in a broader distribution pattern than *HCN4* and includes the ventricular myocardium, but is also moderately expressed in the sinoatrial node[108].

## *Connexins Expressed in Cardiac Conduction Tissue*

Myocardial cells of the heart are electrically connected via gap junctions. Gap junctions consist of two connexons, which are hexamers of transmembrane protein subunits called connexins[111], necessary for electrical and metabolic coupling between cells. In the heart, four major connexins have been identified: Cx40, expressed in fast-conducting cardiac tissues and in the atria[112]; Cx43, expressed in the slower-conducting working myocardium of the atria and ventricles, and in the distal part of the conduction system[71]; Cx45, expressed in slow-conducting pathways, including the sinoatrial node and AV node, and in the myocardium of the primary heart tube[113,114]. Recently, a novel isoform of connexin has been identified, Cx30.2, that is expressed mainly in the conduction system of the heart, predominantly in the sinoatrial node, AV node, and AV bundle[115]. The latter expression patterns are the patterns as observed in adult mouse hearts. However, the expression of connexins in the heart is variable between different species and also varies during the different stages of development[112]. A schematic overview of expression of Cx40, 43, 45, and 30.2 in the adult mouse heart is provided in Fig. 8.

Cx40 can be detected starting from E9.5 in the mouse heart, when it is present first in the primitive atria and primitive left ventricle, later also in the primitive right ventricle, but not in the AV canal and interventricular septum. During further development, together with the development of the specialized CCS, expression becomes restricted to atrial myocytes (but also appears to be present in the right venous valve of the embryonic sinus venosus) and the ventricular conduction system[112,116].

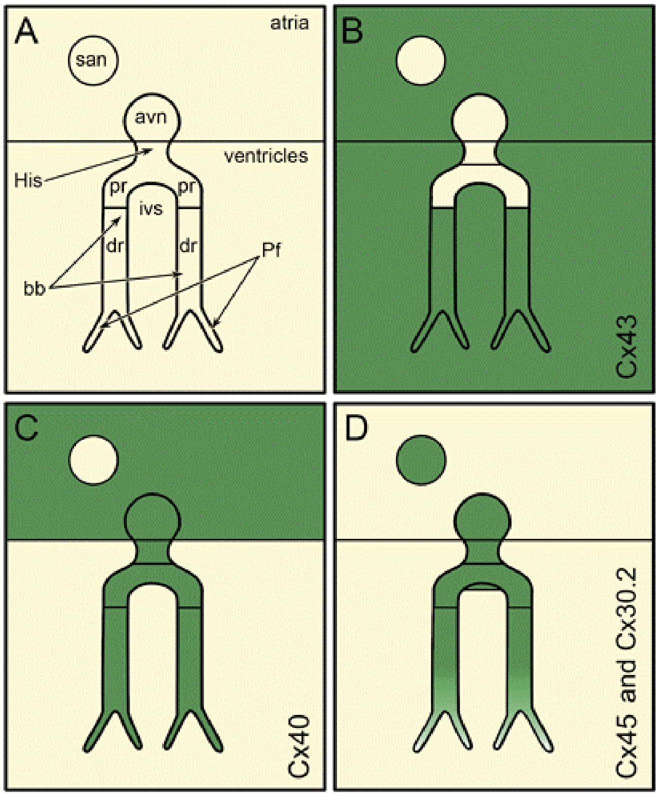

**FIGURE 8.** Schematic overview of the expression of Cx40, 43, 45, and 30.2 in the adult mouse heart. The expression pattern of Cx30.2 largely colocalizes with expression of Cx45[115]. avn: atrioventricular node, bb: bundle branches, dr: distal part of bundle branch, His: His bundle, ivs: interventricular septum, Pf: Purkinje fibers, pr: proximal part of bundle branch, san: sinoatrial node. Adapted from Miquerol et al.[71]

In adult species, Cx40 expression has also been demonstrated in the sinus node in rabbit[117], dog[118], and human[119], and in the AV node of several species, including rabbit[120], mouse[114], and rat[114,121,122].

*Cx40* deficiency results in sinoatrial conduction defects, significant decrease of conduction velocities in the atria, and conduction delay in the His bundle[71]. *Cx40* knock-out mice display an increased incidence of inducible atrial arrhythmias, and significant conduction delay in infra-His and AV nodal conduction[123,124,125,126]. Possibly, *Cx45* compensates partially for the lack of *Cx40* in these mouse models[127].

*Cx43* is expressed in an inverse pattern of Cx40, and is first detected in the primitive ventricle at E9.5 and in the atria at E12.5. At later embryonic stages (E14.5 onward), Cx43 expression increases and is present in the adult ventricular (working) myocytes[128,129]. *Cx43* knock-out mice die at birth because of developmental defects in the pulmonary outflow tract, presumably resulting from defective migration of cardiac neural crest cells to this region[130]. Cardiac-specific deletion of *Cx43* results in sudden cardiac death from spontaneous ventricular arrhythmias at 2 months postnatal, which indicates an important role for Cx43 for maintenance of electrical stability in the heart[131].

Cx45 is expressed already in all compartments of the linear heart tube (E8.5), including the inflow tract, AV canal, and outflow tract. Expression of Cx45 decreases throughout development and in the adult mouse heart; Cx45 is present in the AV node, His bundle, and surrounding the Purkinje fibers[113,114]. *Cx45* knock-out mice demonstrate conduction block and die of heart failure at E10[132].

Cx30.2 decelerates the impulse conduction through the AV node and thus contributes to the slowdown of the impulse propagation through the AV node, which is important in preventing rapid conduction to the ventricles[133,134]. Mice in which the coding region of *Cx30.2* has been replaced by a *lacZ* reporter gene demonstrate a shortening of the QT interval by 25% compared to wild-type mice, due to a significantly accelerated conduction above the level of the His bundle[134].

## DEVELOPMENT OF THE EMBRYONIC ECG

The rhythmic heartbeat, which is characterized by sequential contraction of the atria and ventricles, is coordinated by a complex network of cells throughout the heart, the cardiac pacemaking and conduction system (CCS). In the adult heart, the slow-conducting components of this system are the sinoatrial node and AV node; the fast-conducting elements are the common bundle of His, the right and left bundle branches, and the peripheral Purkinje fibers.

Peristaltic contraction of the tubular heart can be observed as early as 23 days postconception (dpc) in the human (8.5 dpc in mouse), which results in propulsion of blood from the venous to the arterial pole of the heart[8]. Sequential contraction of atria and ventricles can be observed as soon as cardiac looping starts, along with the occurrence of a surface ECG. Although a distinct sinoatrial node primordium can only be detected at E11 in murine hearts[49], pacemaker property is already present in the primitive heart tube in the sinoatrial region at the venous pole of the heart. Interestingly, the earliest pacemaker has been demonstrated at the site of the *left* atrium primordium and during development, shifts toward the right atrial primordium[58,135]. The change in activation pattern from a base-to-apex to the mature apex-to-base pattern, which is needed for efficient ejection of blood from the ventricles in the outflow tracts of the heart, is a consequence of the development of the His-Purkinje system, and reflects the impulse propagation through this rapidly conducting system. This change initiates before ventricular septation is completed and is described to be fully accomplished after septation in the majority of cases[52,99,136]. Recent data from our group show that septation may not be crucial in this process, since electrophysiologic experiments demonstrated premature ventricular base activation to remain present in over half of postseptated embryonic quail hearts until near hatching stages[52].

It is described that the timing of the maturation of the His-Purkinje system may depend on hemodynamic loading, as pressure overload accelerates the timing of the change to an apex-to-base activation pattern, whereas a decreased loading of the embryonic ventricle delays this conversion in ventricular activation sequence[137]. The possible importance of hemodynamics in conduction system development also seems to be demonstrated by the fact that pressure overload of the ventricle results in significantly increased expression of endothelin-converting enzyme 1, a precursor of active endothelin, which is a shear stress–dependent factor involved in the conversion of working cardiomyocytes into conduction system cells, and Cx40-positive Purkinje fibers[138].

In order for the AV conduction axis to become functional, electrical isolation of the atria from the ventricles must occur, except at the site of the AV node/His bundle. However, a typical electrogram characterized by a p-wave reflecting atrial activation, AV delay demonstrated by electrical silence on the ECG, and a QRS-complex reflecting ventricular activation can already be recorded from the embryo at early stages, when the fibrous isolation of the ventricles has not yet been completed (Fig. 9), indicating that functional isolation between atria and ventricles is present before anatomical isolation of the atria from the ventricles has been achieved. On the other hand, as mentioned above, due to persistent accessory myocardial continuities between atrium and ventricle, premature activation of the ventricles can remain present even after septation[52]. In the quail heart, left-sided accessory pathways were less frequently encountered than right-sided pathways, which suggests a developmental time difference in completion of left and right AV ring isolation[52], which is consistent with the relative late development of the right ventricular inlet[105].

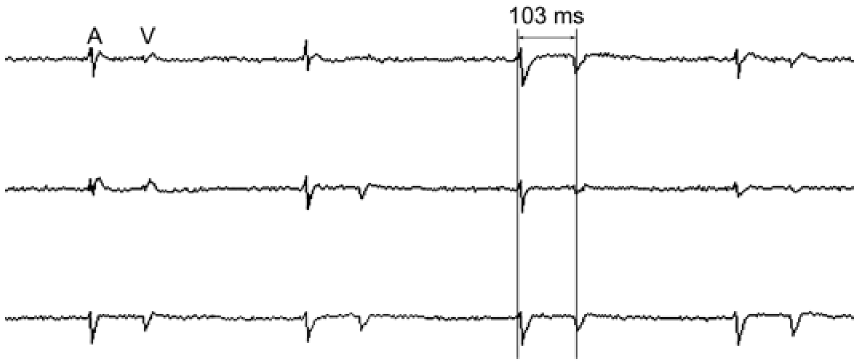

**FIGURE 9.** ECG recording of a murine embryo aged 10.5 days. The cardiac activation is characterized by two deflections representing atrial (A) and ventricular (V) activation, separated by a delay (103 ms).

Neural crest cells may also play an important role in the maturation of cardiac conduction, as neural crest ablation in the chick results in lack of differentiation of a compact lamellar organization by the His bundle and of (electrical) isolation from the working myocardium, and in failure of the conduction system the covert to a mature apex-to-base activation pattern[40]. The role of neural crest cells may largely be inductive, as neural crest cells are present near elements of the CCS during a critical time span before they undergo a process of apoptosis[37].

Next, EPDCs may also play an important role in AV isolation. EPDCs colocalize with periostin in the fibrous heart skeleton, which has been suggested to induce the transformation of myocardium into mesenchyme and fibrous tissue in later stages[36,139,140]. Disturbance of EPDC formation by proepicardial outgrowth inhibition results in reduced periostin expression in the endocardial cushions and AV junction, indicating that EPDCs are local producers of periostin[43,46,141]. Reduced expression of periostin results in disturbed development of fibrous tissue at the AV junction and in persistent AV myocardial connections, resulting in ventricular pre-excitation [36,46, and D.P.Kolditz, A.C. Gittenberger-de Groot, unpublished data].

## SEMANTIC ISSUES REGARDING THE DEFINITION OF THE CARDIAC CONDUCTION SYSTEM

Over the years, several controversies regarding semantics and different applications of definitions have elicited strenuous discussions between researchers in the field of cardiac anatomy and development. One of these issues regards the question of whether it is justified to name the tissues in the embryonic heart that are responsible for the embryonic ECG conduction tissue[142].

Discussions about the definitions of the adult conduction system led Aschoff and Monckeberg in 1910 to describe three prerequisites that must apply to tissues in order to be designated "cardiac conduction tissue". These criteria are (1) cells should be histologically distinct, (2) cells should be able to be followed from section to section in serially prepared tissues, and (3) the specialized cells should be insulated from the working myocardium by sheets of fibrous tissue[143,144]. However, these criteria are not always compelling, as not all three criteria apply to all components of the CCS, such as the tissues of the sinoatrial and AV node, tissues that are generally accepted to be part of the cardiac pacemaking and conducting system.

Also, these adult criteria do not seem to apply to the developing CCS, since the embryonic heart already demonstrates sequential contraction of atria and ventricles regulating blood flow, concomitant with a mature surface ECG, well before the criteria of Aschoff and Monckeberg apply to these tissues.

Furthermore, several molecular markers and functional criteria are now available that help to distinguish working myocardium from myocardium that displays a more specialized phenotype, before fibrous insulation is achieved.

Throughout this article, when we refer to the developing CCS, the entire cardiac pacemaking and conduction system is meant, which thus means not only the nodal tissues, nor only the fast-conduction tissues, but the entire network of nodes, tracts, and fibers responsible for the coordinated, and in some cases, uncoordinated contraction of the heart, which is reflected by the electrical registration on the surface ECG.

## DEVELOPMENT OF THE CARDIAC CONDUCTION SYSTEM IN RELATION TO PUTATIVE SITES OF CLINICAL ARRHYTHMIAS

It is well known from electrophysiological studies that the occurrence of clinical arrhythmias is related to anatomical predilection sites. Clinical mapping studies have demonstrated that ectopic pacemaker foci are preferentially encountered in specific parts of the right and left atrium. In the right atrium, foci are often encountered in sinus venosus–related areas, such as the crista terminalis[145,146], a structure related to the initiation/perpetuation of atrial flutter; and the ostia of the caval veins[147] and coronary sinus[148] as initiators of clinical arrhythmias. In the left atrium, atrial fibrillation has been attributed to arrhythmogenic foci that originate from the pulmonary veins[149]. Furthermore, the interatrial bundle of Bachmann and accessory pathways, such as present in Wolf-Parkinson White (WPW) syndrome and Mahaim tachycardia, are anatomical structures important in cardiac conduction and arrhythmogenesis[150,151]. Moreover, it has been demonstrated that the myocardium at the AV junction itself has specialized properties, and arrhythmias originating from both the tricuspid and mitral junction have been described[152,153]. The question therefore arises as to why these structures, which do not belong to the mature CCS, are able to generate or sustain arrhythmias. An answer to this question may be found in the embryonic development of the CCS. In the following sections, specific anatomical sites that are related to arrhythmogenesis in human are described.

### Internodal Pathways

One of the areas of controversy over the last decades, lasting now for almost a century, is the existence of functional internodal tracts. These internodal tracts have been described to run in the right atrium between the sinoatrial and the AV node[154]. The pathways as originally described by James consist of three cellular tracts, distinguishable from the atrial myocardium based on histological study of atrial sections (stained mostly with Goldner Trichome): a *posterior pathway* along the crista terminalis, an *anterior pathway* that continues to the left atrium via Bachmann's bundle, and a *medial pathway* that runs in the interatrial septum. Specialized Purkinje-like and transitional cells could be demonstrated in these three pathways[154,155,156]. In the embryonic heart, internodal tracts have been distinguished from the atrial myocardium based on the expression pattern of the immunological marker HNK1[55]. In this study, HNK1 was detected in the right venous valve (the putative crista terminalis that will form the boundary between the trabeculated and smooth-walled myocardium of the right atrium), corresponding to the posterior pathway as described by James; in the left venous valve, that in humans becomes incorporated in the interatrial septum; and in an anterior pathway consisting of the septum spurium (the fused anterior right and left venous valves), that could be followed towards the left atrium in a retroaortic position[157]. Recent data of molecular studies also indicate that the embryological sinus venosus is molecularly distinct from the surrounding working atrial myocardium, as similar patterns of expression are observed with the molecular marker *CCS-lacZ*, MinK-*lacZ*, and *Shox2*[86,98,104]. As mentioned earlier, targeted mutation of the *Shox2* gene results in severe hypoplasia of the sinus venosus myocardium, including the sinoatrial nodal region and the venous valves[86].

Although tracts with different histological, immunohistochemical, and molecular characteristics can thus be distinguished in the atria, the functionality of these tracts is yet to be determined. Results of several studies have suggested preferential spread of atrial activation in a fashion that may correspond to these pathways. For instance, optical mapping studies have demonstrated a nonradial spread of intra-atrial conduction in the rat, and the recorded conduction patterns were preferential in a pattern corresponding to the posterior and anterior pathways as described by James[158]. However, whether this preferential conduction in the atria, as is observed in these regions, is due to the presence of specialized cells or is merely an anisotropic organization of tissue[159] remains to be determined. Studies in 1966 and 1967 demonstrated that the administration of elevated levels of potassium induced electrical quiescence of the atrial myocardium, with the exception of cells specifically localized in the areas corresponding the internodal pathways[160,161]. More recently, Racker demonstrated three bundles with unique potential and conduction capacities in dogs, which run between the sinoatrial and AV node, supporting the presence of specialized properties of cells in these areas[162].

## Pulmonary Veins

Since arrhythmogenic capacities have been attributed to the pulmonary veins, these structures have become an important subject of interest, both for those working in the clinical field of electrophysiology and for those working in basic science. In the following sections, a short overview of morphological, molecular, and electrophysiological data in relation to the controversial presence of specialized myocardium at the site of the pulmonary veins is provided.

### *Myocardialization of the Pulmonary Veins: Development of a Myocardial Sleeve*

The arrhythmogenic capacities of the pulmonary veins have been attributed to sleeves of myocardium that surround the pulmonary veins. Anatomical studies describe in detail the length and thickness of the veins[163,164]. In general, the myocardial sleeves surrounding the left superior pulmonary vein are the longest, whereas the sleeves surrounding the right inferior pulmonary vein are shorter, and in some cases absent[163]. These data correspond with the frequency of ectopic foci encountered in clinical mapping studies[149].

The mechanisms of the development of the myocardial sleeves of the pulmonary veins are unresolved. The sleeves could develop due to a process of *myocardialization*, i.e., growth of existing cardiomyocytes into mesenchyme, or *migration* of myocardial cells from the sinoatrial region (now referred to as the posterior heart field) to the pulmonary veins[23]. Although this mechanism may underlie the process of myocardialization of the coronary veins[24], a process of *recruitment* and *differentiation* of cells from the mediastinal mesocardium (the posterior heart field) into cardiomyocytes seems the most likely mechanism behind the second wave of myocardialization responsible for the myocardium formation at the sites of the systemic and pulmonary veins[10,11,14,22,104,165]. In the mouse, this secondary myocardialization of the pulmonary veins has been observed starting at E12.5[23,104,165].

In mouse models where markers of the posterior heart field are deficient, myocardialization of the pulmonary veins is disturbed, as is observed in *Pitx2c*[22] and *podoplanin*-deficient mice (E.A.F. Mahtab, A.C. Gittenberger-de Groot, in preparation).

An interesting question is why these myocardial sleeves possess specialized capacities responsible for the ectopic beats initiating and sustaining clinical tachycardias[149,166]. The next sections describe the results of several morphological and electrophysiological studies that support the presence of specialized characteristics of the myocardium surrounding the pulmonary veins.

## *Morphological Studies Indicative for the Presence of Specialized Conduction Cells in the Pulmonary Veins*

In 1874, attention was drawn to possible independent pacemaking activity of the pulmonary veins by Brunton and Fayrer, who observed independent pulsation of the pulmonary veins in the otherwise mechanical silent hearts of rabbits and cats[167]. In 1986, Masani studied the structure of the myocardial layer surrounding the pulmonary veins in rats, and was able to demonstrate the presence of cells with clear cytoplasm, few myofibrils, and round or oval mitochondria, which resembled sinus node pacemaker cells[168].

More recently, Perez-Lugones et al. identified the presence of P cells, transitional cells, and Purkinje cells in human pulmonary veins[169]. Interestingly, these cells were mainly found in the pulmonary veins of subjects with atrial fibrillation.

It has been hypothesized that deterioration or destruction of the primary pacemaker results in an atrial rhythm originating from these ectopic nodal foci[170]. Next to the pulmonary veins, cells resembling cardiac conduction cells have also been identified in the Eustachian ridge of cats[171] and in Bachmann's bundle in dogs[156].

## *Electrophysiological Studies Performed in Pulmonary Veins*

Several studies demonstrated distinct electrophysiological capacities of pulmonary veins as compared to the atria[172,173,174]. A greater degree of decremental conduction and shorter effective refractory periods have been observed in the myocardial sleeves of the pulmonary veins as compared to the myocardium of the atrium in patients with paroxysmal atrial fibrillation[175,176].

Pulmonary venous cardiomyocytes have distinct electrophysiological properties compared to cardiomyocytes in the left atrium, with a reduced resting membrane potential, action potential amplitude, a smaller phase 0 upstroke velocity, and a shorter duration of the action potential[172]. In accordance with these findings, it has been demonstrated that the myocardium surrounding pulmonary veins has different ionic current properties in comparison with the left atrium. The inward rectifier current is smaller, whereas the delayed rectifier currents are larger in the pulmonary vein than in the left atrium[172]. These results are supported by a study of Chen et al., who distinguished 76% pacemaker cardiomyocytes and 24% nonpacemaker cardiomyocytes in pulmonary veins, with distinct action potentials and ionic current properties[174].

The exact mechanism of the contribution of the pulmonary veins to arrhythmogenicity is still unresolved. Independent spontaneous pacemaker activity has been demonstrated in the pulmonary veins of guineas pigs, rabbits, and cats[177]. More recent reports also support *abnormal automaticity* or *enhanced pacemaker activity* in the pulmonary veins, with or without infusion of medication or pacing maneuvers[177,178,179,180,181]. Furthermore, independent atrial fibrillation has been demonstrated in the pulmonary veins[182].

Moreover, *enhanced triggered after-depolarizations*, sometimes in combination with spontaneous activity, have been supposed as the mechanism responsible for the arrhythmogenicity of the pulmonary veins[183,184,185,186].

The anisotropic arrangement of the myocardium may form the substrate for *re-entry* at this site, which may be important for maintenance of the arrhythmia once it has been initiated[187]. The cellular properties of the pulmonary venous cardiomyocytes mentioned above, including reduced resting membrane potential, action potential amplitude, a smaller phase 0 upstroke velocity, and a shorter duration of the action potential, resulting in shorter refractoriness and slowed conduction, favor the occurrence of re-entry[172,181,188,189].

## Molecular Markers Expressed in Pulmonary Veins

In accordance with findings of different electrophysiological properties of the pulmonary venous myocardium, differences in ion channel subunit expression have been observed in the pulmonary veins compared to the left atrial free wall. These differences include a greater abundance of the rapid-delayed rectifier α-subunit HERG and of the slow-delayed rectifier α subunit KvLQT1, and lower abundance of the inward rectifier subunit Kir2.3, which may underlie the differences in ionic currents observed between pulmonary veins and left atrial cardiomyocytes[190].

The molecular marker CCS-*lacZ* is expressed in the left atrial myocardium surrounding the entrance of the primitive pulmonary vein in early developmental stages (Fig. 10a,b). At later stages, a myocardial sleeve develops surrounding the pulmonary veins (Fig. 10c). Interestingly, this sleeve demonstrates marked CCS-*lacZ* expression[104]. Podoplanin is expressed in the pulmonary veins in a pattern complementary to CCS-*lacZ*. In contrast to atrial myocardium, which strongly expresses Nkx2.5, Nkx2.5 is initially expressed in a only mosaic pattern in the pulmonary veins[10] (Fig. 5), and this area eventually becomes completely Nkx2.5 positive.

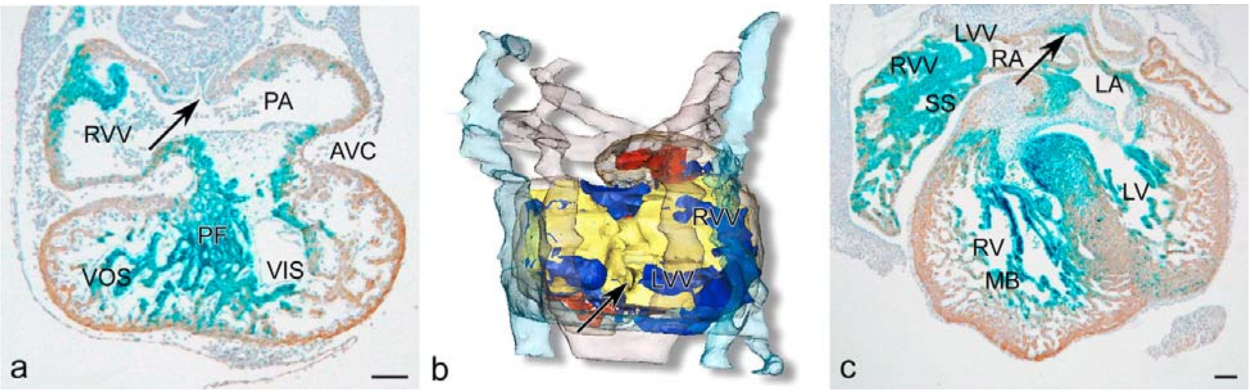

**FIGURE 10.** CCS-*lacZ* expression at the site of the developing pulmonary veins. a: Transverse section of an E10.5 murine embryo, at the level of entrance of the primitive pulmonary vein (arrow), which at this stage does not demonstrate CCS-*lacZ* staining. b: Dorsal view of a 3-D reconstruction of the same embryo. Ventricular myocardium, branchial arch arteries, and cardinal veins are transparent. Color codes — yellow: lumen of the common atrium and the primitive pulmonary vein (PPV); red: lumen of the common ventricle; blue: CCS-*lacZ*–positive myocardium. The arrow points to the primitive pulmonary vein. C: Transverse section of an E13.5 embryo, which now shows marked CCS-*lacZ* expression of the myocardium surrounding the pulmonary vein (arrow). Marked staining is also visible in the right ventricular moderator band (MB). Abbreviations — AVC: atrioventricular canal, LA: left atrium, LV: left ventricle, LVV: left venous valve, PA: primitive atrium, PF: primary fold, RA: right atrium, RV: right ventricle, RVV: right venous valve, SS: septum spurium, VIS: ventricular inlet segment, VOS: ventricular outlet segment. Scale bars — a: 100 µm, c: 200 µm.

Recently, Gudbjartsson et al. reported a strong association between the occurrence of atrial fibrillation and two sequence variants on chromosome 4q25. Interestingly, both variants are located in the genome adjacent to *Pitx2c*[191], which is highly expressed in the posterior heart field on the left side, surrounding the pulmonary vein prior to and during formation of the pulmonary myocardium and later becomes confined to the pulmonary myocardium[22,192].

## AV Junction: Accessory Pathways/Mahaim Fibers

In early embryonic stages, atrial and ventricular myocardium is continuous through the myocardium of the AV canal. In normal adult cardiac conduction, the AV conduction axis is the only functional AV conduction tract. AV re-entrant tachycardias are based on the presence of accessory myocardial bundles connecting atrial and ventricular tissue, thus bypassing the insulating function of the AV groove. The most well known is the bundle of Kent, present in the WPW syndrome[193]. As was demonstrated in chick, accessory AV myocardial continuities may persist in the embryo until late stages, causing premature activation of the ventricles even after septation[52].

Moreover, several arrhythmias have been described in literature that originate from the tricuspid and mitral junction[152,153]. AV junctional cells surrounding both the tricuspid and mitral annuli resemble nodal cells in their cellular electrophysiology[194].

A special form of re-entrant tachycardia is Mahaim tachycardia, during which antidromic re-entrant tachycardia occurs over an accessory bundle with AV node–like conduction properties. The proximal insertion often localized to the lateral, anterolateral, or posterolateral tricuspid annulus and distal insertion into the right ventricular free wall or the right bundle branch[195]. To date, there are two mouse models for WPW syndrome. Mutations in the gene *PRKAG2* (that encodes the γ-2 subunit of the AMP-activated protein kinase) have been observed in patients with WPW syndrome[196]. Mice that carry a mutation in the *PRKAG2* gene display ventricular pre-excitation and a phenotype identical to humans with a familial form of ventricular pre-excitation[197]. Patel et al. demonstrated the *postnatal* development of myocardial connections through the annulus fibrosus of the AV valves in mice overexpressing the *PRKAG2* mutation[198]. The findings in these models seem to be associated with cardiac hypertrophy, accumulation of excessive amounts of cardiac glycogen, and disruption of the annulus fibrosus by glycogen-filled cardiomyocytes[199].

Furthermore, specific deletion in the AV canal of the gene *ALK3,* coding for the type 1a receptor for bone morphogenetic proteins in the AV canal during development, causes ventricular pre-excitation, indicating an important role of this gene in proper AV junction development[200,201].

As was mentioned earlier, epicardial inhibition studies demonstrate reduced periostin expression at the AV junction, resulting in disturbed development of fibrous tissue at the AV junction, persistent AV myocardial connections with resulting ventricular pre-excitation, which may be an mechanism explaining WPW-syndrome[36,46, and D.P. Kolditz, A.C. Gittenberger-de Groot, unpublished data].

Data derived from the CCS-*lacZ* mouse demonstrate that the occurrence of Mahaim fibers may be related to the embryonic development of the right ventricular inflow tract. The development of the right atrial/right ventricular connection and concomitant outgrowth of the right ventricle results in a division of the CCS-*lacZ*-positive tissue of the primary fold that originally separated the primitive left and right ventricles. This division results in the development of the right ventricular moderator band (Fig. 10c) that forms a right-sided AV continuity, similar to a Mahaim fiber. Electrophysiological experiments supported the presence of a conducting right-sided AV pathway[105].

## CONCLUSION

Although the exact mechanisms of CCS development are still to be unraveled, it has become clear that the development of the CCS is a multifactorial process, in which multiple factors are involved and interact. Based on the expression patterns of several known markers, the developing conduction system seems to be more extensive than the definitive adult CCS. Furthermore, areas of "primitive" conduction system correlate with predilection sites for the occurrence of clinical arrhythmias in adults. We hypothesize that either embryonic remnants, or the re-expression of an embryonic phenotype, may explain this correlation. The processes that induce cardiomyocytes to acquire a cardiac conduction phenotype have not been elucidated. Recent data support a contribution of cells from the posterior heart field to the formation of the CCS.

## ACKNOWLEDGMENTS

Jan Lens and Ron Slagter are greatly acknowledged for their help in preparing the figures for the manuscript.
